# Supplementary material for: The Impostor Phenomenon in the Nutrition and Dietetics Profession: An Online Cross-Sectional Survey
Source: Int J Environ Res Public Health. 2022 May 3;19(9):5558. doi: 10.3390/ijerph19095558 (PMC9099444; doi:10.3390/ijerph19095558)
Supplement: Supplementary file 1 [file ijerph-19-05558-s001.zip › ijerph-1697045-supplementary.pdf]

International Journal of
Environmental Research and Public Health

MDPI

Table S1. Examples of Prevalence of Impostor Phenomenon within Various Health-Related Professions

Reference	Type of Professional (e.g., Nurses, Pharmacists, Medical Students)	Sample Size	Survey Used	Prevalence of IP and Cutoff Used
Ares (2018)[1]	Clinical nurse specialists	68	CIPS	20.5% (CIPS Score ≥62)
Barr-Walker et al (2019)[2]	Health science librarians	703	HIPS	14.5% (HIPS Score ≥42)
Holliday et al (2020)[3]	Medical and dental students	485	CIPS	18% (CIPS Score ≥80)
Jacobs and Sasser (2021)[4]	Nursing students	150	CIPS	48.7% (CIPS Score ≥61)
Kimball et al (2020)[5]	Chiropractic students	406	CIPS	39% (CIPS Score ≥62)
Levant et al (2020)[6]	Third year medical students	112	CIPS	51% (CIPS Score ≥62)
Paladugu et al (2021)[7]	Hospitalists	71	CIPS	33.8% (CIPS Score ≥60)
Schmulian et al (2020)[8]	Graduate allied health students	72	YIS	37.5% (YIS Score ≥5)
Shreffler et al (2021)[9]	Medical students	233	CIPS	42.1% (CIPS Score ≥61)
Sullivan and Ryba (2020)[10]	Pharmacy residents	720	CIPS	57.5% (CIPS Score ≥62)

Abbreviations: CIPS, Clance Impostor Phenomenon Scale; HIPS, Harvey Impostor Phenomenon Scale; YIS, Young Impostor Scale

Table S1 References

1. Ares, T.L. Role transition after clinical nurse specialist education. *Clinical Nurse Specialist* **2018**, *32*, 71-80.
2. Barr-Walker, J.; Bass, M.B.; Werner, D.A.; Kellermeyer, L. Measuring impostor phenomenon among health sciences librarians. *J Med Libr Assoc* **2019**, *107*, 323.
3. Holliday, A.M.; Gheihman, G.; Cooper, C.; Sullivan, A.; Ohyama, H.; Leaf, D.E.; Leaf, R.K. High prevalence of imposterism among female Harvard medical and dental students. *J Gen Intern Med* **2020**, *35*, 2499-2501.
4. Jacobs, M.D.; Sasser, J.T. Impostor Phenomenon in Undergraduate Nursing Students: A Pilot Study of Prevalence and Patterns. *J Nurs Educ* **2021**, *60*, 329-332.
5. Kimball, K.A.; Roecker, C.B.; Hoyt, K. Impostor phenomenon among US chiropractic students. *J Chiropr Educ* **2020**.
6. Levant, B.; Villwock, J.A.; Manzardo, A.M. Impostorism in third-year medical students: an item analysis using the clance impostor phenomenon scale. *Perspect Med Educ* **2020**, *9*, 83-91.
7. Paladugu, S.; Wasser, T.; Donato, A. Impostor syndrome in hospitalists-a cross-sectional study. *J Community Hosp Intern Med Perspect* **2021**, *11*, 212-215.
8. Schmulian, D.; Redgen, W.; Fleming, J. Impostor syndrome and compassion fatigue among graduate allied health students: A pilot study. *Focus on Health Professional Education: A Multi-disciplinary Journal* **2020**, *21*, 1-14.
9. Shreffler, J.; Weingartner, L.; Huecker, M.; Shaw, M.A.; Ziegler, C.; Simms, T.; Martin, L.; Sawning, S. Association between characteristics of impostor phenomenon in medical students and step 1 performance. *Teach Learn Med* **2021**, *33*, 36-48.
10. Sullivan, J.B.; Ryba, N.L. Prevalence of impostor phenomenon and assessment of well-being in pharmacy residents. *American Journal of Health-System Pharmacy* **2020**, *77*, 690-696.
